# Benefit of Later-Time-Point PET Imaging of HER3 Expression Using Optimized Radiocobalt-Labeled Affibody Molecules

**DOI:** 10.3390/ijms21061972

**Published:** 2020-03-13

**Authors:** Sara S. Rinne, Charles Dahlsson Leitao, Zahra Saleh-nihad, Bogdan Mitran, Vladimir Tolmachev, Stefan Ståhl, John Löfblom, Anna Orlova

**Affiliations:** 1 Department of Medicinal Chemistry, Uppsala University, 751 83 Uppsala, Sweden; sara.rinne@ilk.uu.se (S.S.R.); zahra-zajnab@hotmail.com (Z.S.-n.); bogdan.mitran@ilk.uu.se (B.M.); 2 Department of Protein Science, School of Engineering Sciences in Chemistry, Biotechnology and Health, KTH Royal Institute of Technology, 106 91 Stockholm, Sweden; chdl@kth.se (C.D.L.); ssta@kth.se (S.S.); lofblom@kth.se (J.L.); 3 Department of Immunology, Genetics and Pathology, Uppsala University, 751 85 Uppsala, Sweden; vladimir.tolmachev@igp.uu.se; 4 Research Centrum for Oncotheranostics, Research School of Chemistry and Applied Biomedical Sciences, Tomsk Polytechnic University, 634050 Tomsk, Russia; 5 Science for Life Laboratory, Uppsala University, 752 37 Uppsala, Sweden

**Keywords:** HER3, PET, gallium-68, radiocobalt, cobalt-55, affibody, NOTA, NODAGA, DOTA, DOTAGA

## Abstract

HER3-binding affibody molecules are a promising format for visualization of HER3 expression. Cobalt-55, a positron-emitting isotope, with a half-life of 17.5 h, allows for next-day imaging. We investigated the influence of the charge of the radiocobalt–chelator complex on the biodistribution of anti-HER3 affibody molecule (HE)_3_-Z_HER3_ and compared the best radiocobalt-labeled variant with a recently optimized gallium-labeled variant. Affibody conjugates (HE)_3_-Z_HER3_-X (X = NOTA, NODAGA, DOTA, DOTAGA) were labeled with [^57^Co]Co (surrogate for ^55^Co). Affinity measurements, binding specificity and cellular processing were studied in two HER3-expressing cancer cell lines. Biodistribution was studied 3 and 24 h post-injection (pi) in mice with HER3-expressing BxPC-3 xenografts and compared to [^68^Ga]Ga-(HE)_3_-Z_HER3_-NODAGA. Micro-single-photon emission tomography/computed tomography (microSPECT/CT) and micro-positron emission tomography/computed tomography (microPET/CT) imaging was performed 3 and 24 h pi. Stably labeled conjugates bound to HER3 with subnanomolar affinity. [^57^Co]Co-(HE)_3_-Z_HER3_-DOTA had the best tumor retention and a significantly lower concentration in blood than other conjugates, leading to superior tumor-to-blood and tumor-to-liver ratios 24 h pi. Compared to [^68^Ga]Ga-(HE)_3_-Z_HER3_-NODAGA 3 h pi, [^57^Co]Co-(HE)_3_-Z_HER3_-DOTA provided superior imaging contrast in liver 24 h pi. Concluding, the composition and charge of the [^57^Co]Co–chelator complex influenced the uptake in tumors and normal tissue. [^57^Co]Co-(HE)_3_-Z_HER3_-DOTA provided the best imaging properties among the cobalt-labeled conjugates. Delayed imaging of HER3 expression with [^57^Co]Co-(HE)_3_-Z_HER3_-DOTA improved imaging contrast compared to early-time-point imaging with [^68^Ga]Ga-(HE)_3_-Z_HER3_-NODAGA.

## 1. Introduction

Upregulation of the human epidermal growth factor receptor type 3 (HER3) is associated with the progression of several solid cancers, such as prostate, breast, pancreatic or colorectal cancer [1,2,3]. HER3 activates the potent PI3K/AKT/mTor signaling pathway, affecting cell proliferation and survival [1], and upregulation of HER3 can be a bypass mechanism for signaling loss of other HER-family members due to HER-targeted therapy [4,5]. Co-expression of HER3 is, therefore, considered a cause for the development of therapy resistance, which has, for instance, been documented for the tyrosine kinase inhibitors (TKIs) lapatinib and gefitinib, targeting epidermal growth factor receptor (EGFR) and HER2 [6,7,8]. Thus, inhibition of HER3-mediated signaling might have potential to overcome therapy resistance [4,9] and monitoring of HER3 expression could, therefore, aid strategic decision making for cancer therapy.

Radionuclide molecular imaging with positron emission tomography (PET) or single-photon emission tomography (SPECT) is a promising non-invasive and repeatable alternative to traditional biopsies for molecular profiling of cancer, allowing for detection of target expression as well as treatment monitoring. HER3 imaging is challenging due to the relatively low overexpression in malignant lesions and moderate endogenous expression in healthy tissue and potential metastatic sites, especially the liver. Radiolabeled antibodies and antibody fragments have been suggested for imaging of HER3-expression [10,11,12]. For example, the anti-HER3 antibody ^89^Zr-lumretuzumab provided good imaging contrast 4–7 days after injection and enabled quantification of the uptake in HER3 positive tumors in a small clinical study. However, only in non-hepatic cancer lesions [13]. A major drawback of radiolabeled antibodies for imaging is the long residence time in blood and their hepatobiliary elimination pathway.

Besides HER3-targeting antibodies and antibody fragments, the use of an affibody molecule, a type of engineered scaffold protein (ESP) [14], is a promising and potentially favorable approach for the detection of HER3 expression [15,16]. Affibody molecules are derived from the Z-domain of staphylococcal protein A and are three-helical proteins consisting of 58 amino acids. The binding motif consists 13 amino acids located on helices 1 and 2. By randomization of these amino acids, affibody molecules have been designed against a number of different targets (e.g., EGFR, HER2, IGF1-R) [17,18]. The smaller size of affibody molecules (7–8 kDa) and their fast pharmacokinetics increases extravasation and tumor penetration, reduces enhanced permeability and retention (EPR) effects and might, therefore, improve imaging contrast compared to antibodies and their derivatives [17,18,19]. Typically, a high contrast is obtained on the same day as the injection, as early as three to four hours post-injection (pi). We and others have previously shown the feasibility of anti-HER3 affibody molecules for imaging of HER3 expression with different radioisotopes for PET and SPECT [16,20,21,22].

Generally, PET is preferred over SPECT because of higher sensitivity and possibility for quantification. Gallium-68-PET has been very successful in preclinical and clinical settings for imaging of different molecular targets [23]. Furthermore, the gallium-68-labeled anti-HER2 affibody ABY-025 has shown clinical success for imaging of HER2-positive primary tumors and metastases shortly after injection [24]. Compared to HER2, the level of HER3 overexpression in cancer cells is low [25] and uptake in normal tissue complicates image interpretation. However, it was previously observed that uptake of HER3 affibody molecules in normal organs decreases over time, while tumor-associated activity cleared more slowly [26,27]. Therefore, HER3 imaging contrast could potentially benefit from delayed imaging (up to 24 h pi), which was previously observed for indium-111-labeled anti-HER3 affibody molecules [26] and for radiolabeled anti-HER1 (EGFR) affibody molecules [28,29,30]. For PET imaging of HER3 expression, ^18^F- and ^68^Ga-labeled Z_HER3_ affibody molecules were able to visualize tumor uptake already 1 and 3 h pi in rodents. However, the tumor-to-liver contrast did not exceed 1 [21,31]. Unfortunately, these two commonly used PET isotopes have short half-lives (t_1/2_(^18^F) = 110 min, t_1/2_(^68^Ga) = 68 min) and are not suitable for next-day imaging.

The long-lived PET isotope zirconium-89 (t_1/2_ = 78.4 h) has been used for preclinical and clinical imaging of HER3 expression in combination with antibodies, and nanobodies [10,13,32]. Recently, a HER3-targeting affibody molecule was labeled with ^89^Zr and used to detect changes in HER3 expression in response to treatment of breast cancer xenografts with an HSP90 inhibitor [22]. However, the fast kinetics of affibody molecules would also allow the use of PET isotopes with intermediate half-lives (shorter than ^89^Zr but longer than ^18^F and ^68^Ga). The use of such radioisotopes would also lower the radiation dose burden to the patients compared to ^89^Zr. Cobalt-55 is a somewhat more unconventional PET isotope, with a half-life of 17.5 h. It can be produced with a medical cyclotron; preclinical evaluation of cobalt-55-labeled tracers targeting PSMA and GRPR, as well as clinical application of cobalt-55 to study brain injuries has been reported [33,34,35].

An initial study of a radiocobalt-labeled affibody Z_HER3_-NOTA showed promising results, reporting for the first time a tumor-to-liver ratio above 1 [27]. Cobalt participates in radiolabeling in a divalent state, thus decreasing the charge of the metal–chelator complex by 1 compared to similar complexes with trivalent metals, such as gallium-68 or indium-111. It was hypothesized that an increased negative charge can reduce unspecific hepatic uptake and, therefore, enhance the tumor-to-liver contrast [27]. This hypothesis was further supported by comparing the effect of differently charged radiometal-chelator complexes on the biodistribution of Z_HER3_ labeled with ^68^Ga and ^111^In and HER2-targeting affibody molecules [26,31,36].

The present study had two aims. Firstly, to evaluate whether the imaging properties of (HE)_3_-Z_HER3_ might be improved by modification of the radiocobalt–chelator complex. Secondly, to investigate the potential benefit of using cobalt-55 as an option for later-time-point PET imaging of HER3 expression instead of imaging with gallium-68 shortly after injection. We, therefore, studied the in vitro and in vivo properties of four different variants of (HE)_3_-Z_HER3_ (further denoted as (HE)_3_-Z_HER3_-X, with X = NOTA, NODAGA, DOTA, DOTAGA) labeled with cobalt-57 and compared the most promising radiocobalt variant with ^68^Ga-(HE)_3_-Z_HER3_-NODAGA. Cobalt-57 is a convenient surrogate for cobalt-55 [35,37] due to its commercial availability and long half-life (272 d). A potential drawback could be different metal impurities for ^55^Co and ^57^Co, which may require re-optimization of the labeling procedure.

## 2. Results

### 2.1. Radiolabeling and Stability Assessment

**Figure 1:**
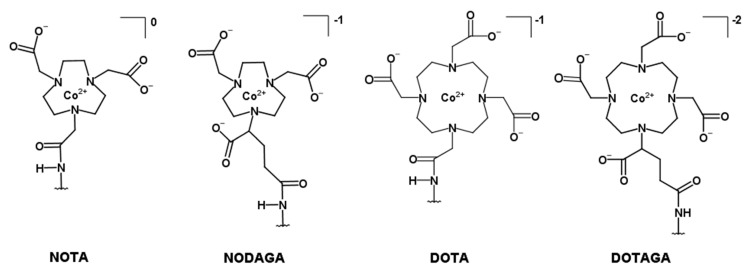
Structural overview of the macrocyclic chelators conjugated to the C-terminus of the HER3-targeting affibody molecule (HE)_3_-Z_08698_ via a C-terminal cysteine (further denoted (HE)_3_-Z_HER3_-X, with X = NOTA (1-(1,3-carboxypropyl)-4,7-carboxymethyl-1,4,7-triazacyclononane), NODAGA (1,4,7-triazacyclononane-N,N′,N″-triacetic acid), DOTA (1,4,7,10-tetraazacyclododecane-1,4,7,10-tetraacetic acid), DOTAGA (1,4,7,10-tetraazacyclododecane, 1-(glutaric acid)-4,7,10-triacetic acid)).

The production and purification of the different affibody molecules (HE)_3_-Z_HER3_-NOTA, (HE)_3_-Z_HER3_-NODAGA, (HE)_3_-Z_HER3_-DOTA, and (HE)_3_-Z_HER3_-DOTAGA are described in Dahlsson Leitao et al. [31]. An overview of the different chelators is depicted in Figure 1.

All new variants were labeled with ^57^Co, with almost quantitative radiochemical yield determined by instant thin-layered liquid chromatography (ITLC) (Table 1, Figure S1). The labeled conjugates were stable in PBS and in human serum determined by ITLC. [^68^Ga]Ga-(HE)_3_-Z_HER3_-NODAGA was labeled with high radiochemical yield (89 ± 1%, determined by ITLC) and radiochemical purity exceeding 98% (ITLC) after purification with size-exclusion chromatography.

### 2.2. In Vitro Characterization of [^57^Co]Co-(HE)_3_-Z_HER3_-X

**Figure 2:**
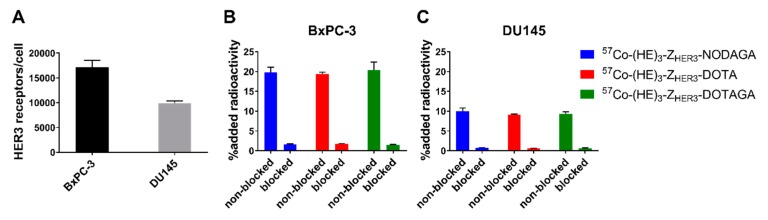
Receptor quantification and in vitro specificity. (**A**) HER3 expression was quantified for BxPC-3 (n = 2) and DU145 (n = 2) cells by incubation with [57Co]Co-(HE)3-ZHER3-NOTA until saturation. For the in vitro specificity test in (**B**) BxPC-3 and (**C**) DU145 cells, binding to HER3 was inhibited by addition of 50 nM HER3 binding affibody in the blocked groups. Specificity data is presented as the average of three dishes ± SD.

HER3-expressing cell lines BxPC-3 and DU145 were used for the in vitro characterization. The receptor density was 17180 ± 1369 receptors/cell for BxPC-3 cells and 9931 ± 430 receptors/cells for DU145 cells (Figure 2A).

Pre-saturation of HER3 receptors significantly reduced (90–97% reduction) the binding of all conjugates to HER3-expressing cells (Figure 2B,C). Thus, binding of [^57^Co]Co-(HE)_3_-Z_HER3_-X conjugates was HER3 specific. Binding specificity of [^57^Co]Co-(HE)_3_-Z_HER3_-NOTA was previously demonstrated [27].

Binding kinetics were measured in real time on BxPC-3 cells (Figure S2). The K_D_ was in the subnanomalar rage for all conjugates without significant differences between the conjugates (Table 2) and without significant differences in association and dissociation rates.

**Figure 3:**
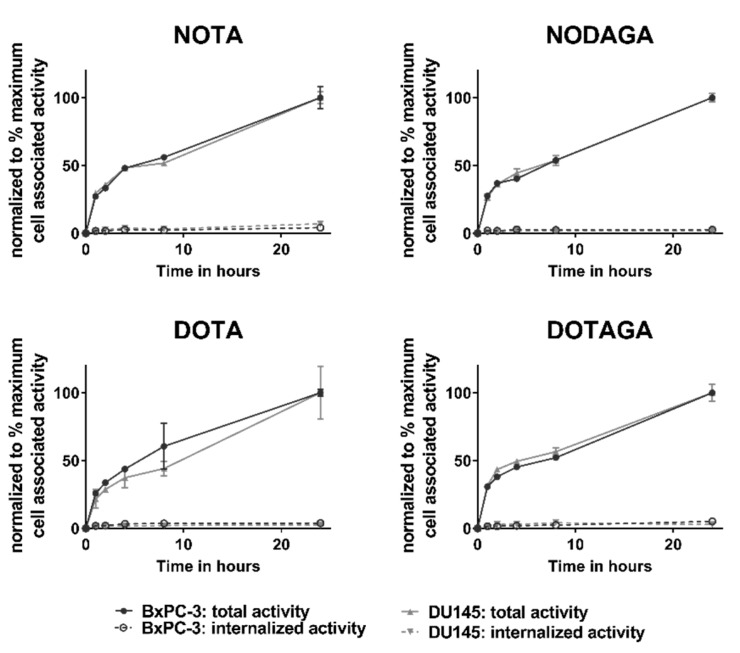
Internalization assay in BxPC-3 cells (black) and DU145 (grey) cells. Fractions of internalized and cell-associated activity of [^57^Co]Co-(HE)_3_-Z_HER3_-X are shown as normalized to the maximum cell-associated activity. Each data point is the average of three dishes ± SD, *n* = 3.

To study the internalization properties of the labeled conjugates, BxPC-3 and DU145 cells were continuously incubated with [^57^Co]Co-(HE)_3_-Z_HER3_-X for 24 h (Figure 3). No differences were observed between the conjugates and cell lines. Binding to cells was rapid and total cell-bound activity continued to increase up to 24 h. The fraction of internalized activity did not exceed 5% for any conjugate at the end of the observation period.

### 2.3. In Vivo Evaluation

**Figure 4:**
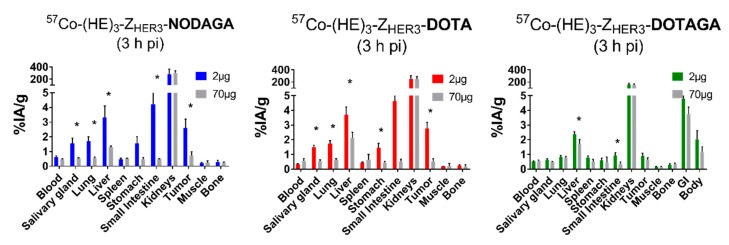
In vivo specificity. Tumor-bearing female Balb/c nu/nu mice were injected with 2 µg of labeled conjugates or excess amount (70 µg) of non-labeled anti-HER3 affibody molecules. Data presented as the average ± SD of *n* = 3–4 animals/group. * Indicates significant difference *p* < 0.05 between the 2 and 70 µg groups.

In vivo experiments were performed on female Balb/c nu/nu mice bearing HER3-expressing BxPC-3 xenografts injected with 2 µg [^57^Co]Co-(HE)_3_-Z_HER3_-X. The results from the in vivo experiments are shown in Table 3 and Table 4, Figure 4.

[^57^Co]Co-(HE)_3_-Z_HER3_-X accumulated in tumors and mErbB3-expressing organs (salivary glands, lungs, liver, stomach, small intestine). Increasing the injected protein dose to 70 µg significantly reduced the uptake of [^57^Co]Co-(HE)_3_-Z_HER3_-NODAGA and [^57^Co]Co-(HE)_3_-Z_HER3_-DOTA in tumors and mErbB3-expressing organs (Figure 4). In the case of [^57^Co]Co-(HE)_3_-Z_HER3_-DOTAGA, the excess amount of protein resulted in a significant decrease in uptake in liver and small intestine. However, this was less pronounced than for the other [^57^Co]Co-(HE)_3_-Z_HER3_-X conjugates. No significant decrease in uptake was observed in tumors in the case of [^57^Co]Co-(HE)_3_-Z_HER3_-DOTAGA. In vivo specificity of [^57^Co]Co-(HE)_3_-Z_HER3_-NOTA was previously demonstrated [27].

The biodistribution of the new conjugates was comparable with [^57^Co]Co-(HE)_3_-Z_HER3_-NOTA in tumor-bearing mice 3 and 24 h pi (Table 3). As expected for affibody molecules, the [^57^Co]Co-(HE)_3_-Z_HER3_-X conjugates were eliminated via the renal pathway with high degree of activity accumulation in the kidneys. Already at 3 h pi, the blood activity concentration of all [^57^Co]Co-(HE)_3_-Z_HER3_-X variants was below 1% ID/g and decreased further after 24 h. The blood activity concentration of [^57^Co]Co-(HE)_3_-Z_HER3_-NOTA was the highest and [^57^Co]Co-(HE)_3_-Z_HER3_-DOTA was the lowest at both time points. Tumor uptake was influenced by the different cobalt–chelator complexes. NODAGA- and DOTA-conjugated variants had significantly higher uptake in tumors than NOTA- and DOTAGA-conjugated variants. Tumor uptake of [^57^Co]Co-(HE)_3_-Z_HER3_-DOTAGA was below 1% ID/g, the lowest among the tested conjugates. After 24 h, tumor uptake of [^57^Co]Co-(HE)_3_-Z_HER3_-NODAGA reduced by almost 2-fold. The other variants did not show a significant decrease in tumor uptake between 3 and 24 h pi.

Overall, [^57^Co]Co-(HE)_3_-Z_HER3_-NODAGA and [^57^Co]Co-(HE)_3_-Z_HER3_-DOTA had a similar uptake to the other conjugates in non-expressing organs and tissues (blood, muscle, bone). However, higher uptake was observed in HER3-expressing organs, which also correlated with higher uptake in tumors. The uptake in normal organs and tissues, such as lung, liver, stomach, and small intestine, significantly decreased with time.

Generally, tumor-to-organ ratios tendended to to be higher at 24 h pi. However, there was no significant difference in tumor-to-non-tumor ratios for [^57^Co]Co-(HE)_3_-Z_HER3_-NODAGA between 3 and 24 h pi. At 3 h, [^57^Co]Co-(HE)_3_-Z_HER3_-NOTA had the highest tumor-to-liver ratio and [^57^Co]Co-(HE)_3_-Z_HER3_-DOTA the highest tumor to blood ratio (Table 4). Most notably, tumor-to-liver-ratio of [^57^Co]Co-(HE)_3_-Z_HER3_-DOTA doubled after 24 h. Overall, [^57^Co]Co-(HE)_3_-Z_HER3_-DOTA had the highest tumor-to-blood, -liver and -muscle ratios among all cobalt-labeled variants at 24 h.

**Figure 5:**
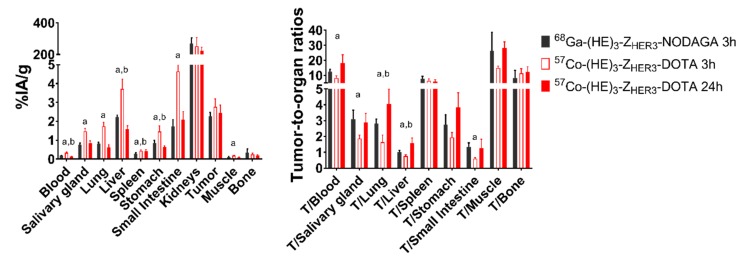
Comparison of the biodistribution of [^57^Co]Co-(HE)_3_-Z_HER3_-DOTA (3 and 24 h pi) and [^68^Ga]Ga-(HE)_3_-Z_HER3_-NODAGA (3 h pi) in BxPC-3 xenografted Balb/c nu/nu mice. Data is presented as the average ± SD of *n* = 4 animals per group. Statistical significance (*p* < 0.05) between ^a^ [^68^Ga]Ga-(HE)_3_-Z_HER3_-NODAGA and [^57^Co]Co-(HE)_3_-Z_HER3_-DOTA 3 h pi and ^b^ [^68^Ga]Ga-(HE)_3_-Z_HER3_-NODAGA and [^57^Co]Co-(HE)_3_-Z_HER3_-DOTA 24 h pi was determined with unpaired, two-tailed t-test. Numerical data is available in Table S1.

The biodistribution of [^68^Ga]-Ga-(HE)_3_-Z_HER3_-NODAGA 3 h pi was directly compared with [^57^Co]Co-(HE)_3_-Z_HER3_-DOTA 24 h pi in the same batch of Balb/c nu/nu mice bearing BxPC-3 xenografts (Table S1, Figure 5). Data for [^68^Ga]-Ga-(HE)_3_-Z_HER3_-NODAGA was in agreement with previously published data [31]. Comparing [^68^Ga]Ga-(HE)_3_-Z_HER3_-NODAGA at 3 h pi with [^57^Co]Co-(HE)_3_-Z_HER3_-DOTA at 24 h pi, the cobalt-labeled variant had significantly lower activity concentration in blood, liver and stomach, and slightly higher uptake in tumor. However, the difference in tumor was non-significant.

At 3 h pi, the tumor-to-blood, tumor-salivary gland, tumor-lung, tumor-liver and tumor-small intestine ratios were higher for [^68^Ga]Ga-(HE)_3_-Z_HER3_-NODAGA than for [^57^Co]Co-(HE)_3_-Z_HER3_-DOTA 3 h pi. However, because tumor-to-organ ratios of [^57^Co]Co-(HE)_3_-Z_HER3_-DOTA increased with time, the tumor-to-liver and tumor-to-lung ratios of [^57^Co]Co-(HE)_3_-Z_HER3_-DOTA at 24 h were significantly higher than for [^68^Ga]Ga-(HE)_3_-Z_HER3_-NODAGA at 3 h. Tumor-to-blood and tumor-to-bone ratios for [^57^Co]Co-(HE)_3_-Z_HER3_-DOTA were also higher. However, the differences were non-significant. There was no difference in the remaining organs and tissues (Figure 5).

### 2.4. Imaging

**Figure 6:**
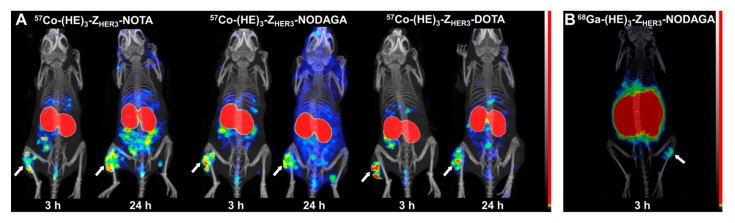
(**A**) Micro-single-photon emission tomography/computed tomography (microSPECT/CT) imaging of [^57^Co]Co-(HE)_3_-Z_HER3_-X at 3 and 24 h pi in mice bearing HER3-expressing BxPC-3 xenografts. [^57^Co]Co-(HE)_3_-Z_HER3_-DOTAGA was excluded from the imaging study because of unfavorable biodistribution prolife. (**B**) Micro-positron emission tomography/computed tomography (microPET/CT) imaging of [^68^Ga]Ga-(HE)_3_-Z_HER3_-NODAGA 3 h pi in a mouse with HER3-expressing BxPC-3 xenograft. Images are displayed as maximum intensity projections (MIP). White arrows indicate the HER3-expressing BxPC-3 xenografts.

Results from microSPECT imaging for [^57^Co]Co-(HE)_3_-Z_HER3_-NOTA, [^57^Co]Co-(HE)_3_-Z_HER3_-NODAGA and [^57^Co]Co-(HE)_3_-Z_HER3_-DOTA at 3 and 24 h pi are displayed in Figure 6. Uptake in tumors and HER3-expressing organs and kidneys could be visualized clearly, and images resembled the results from the biodistribution studies.

## 3. Discussion

HER3 has evolved into an important molecular target in cancer. We and others have previously investigated the potential of radiolabeled affibody molecules as HER3-targeted imaging agents for PET and SPECT [15,16,20,21,22,27]. It has been shown that an increase in the negative charge of the radionuclide–chelator complex can improve tumor-to-liver contrast for affibody molecules [26,36]. In addition, retention of activity in tumors is better than in normal tissues for several types of imaging probes. Thus, imaging at later time points enables further increase of imaging contrast, and hence sensitivity, even for imaging probes with rapid in vivo kinetics and tumor targeting, such as bombesin analogues [38], somatostatin analogues [39], single chain variable fragments (scFv) [40], and affibody molecules [41]. Later-time-point imaging has been shown to improve the imaging quality of HER3-targeting affibody molecules [20,26,27]. The use of PET for imaging provides better sensitivity, spatial resolution and accuracy of quantification, and therefore a positron-emitting label is desirable. The use of a long-lived positron emitter (e.g., ^89^Zr) in combination with efficient reabsorption of affibody molecules in kidney would result in an appreciably elevated dose burden to patients. Therefore, the use of positron-emitting nuclides permitting next-day imaging seems to be a reasonable compromise. A list of such nuclides with half-lives between 9 and 20 h is provided in Table 5. All these nuclides can be produced by low-energy cyclotrons using isotopically enriched targets.

The use of the radiohalogen ^76^Br would be associated with a non-residualizing label, and our latest study showed that this type of label is suboptimal for affibody-mediated HER3 imaging [42]. The chemistry of labeling with ^90^Nb has not been established yet. For ^64^Cu, a low positron yield and an appreciable β^−^ branching ratio is of concern. Among other radiometals, ^55^Co has the lowest positron energy, which is favorable for imaging resolution, and the branching ratio of the positron decay is the highest among the positron emitting nuclides. Previous studies have demonstrated that radiocobalt forms stable complexes with macrocyclic chelators, such as DOTA, NOTA, DOTAGA and NODAGA [37,39]. This makes it a promising candidate for later-time-point PET imaging.

Based on this, the first aim of this paper was to select the chelator providing the most favorable properties for PET imaging of HER3 expression using radiocobalt. The second aim of this study was to investigate the potential benefit of using cobalt-55 for later-time-point PET imaging compared to early-time-point imaging with the well-established PET isotope gallium-68.

A panel of four affibody molecules conjugated to different macrocyclic chelators via a C-terminal cysteine ((HE)_3_-Z_HER3_-X, X = NOTA, NODAGA, DOTA, DOTAGA) was labeled with cobalt-57 (t_1/2_ = 272 d, convenient photons with energies of 122 keV (85%), 136 keV (11%)) as surrogate for cobalt-55. Labeling did not affect the ability of the radioconjugates to bind HER3 specifically with subnanomolar affinity. The different levels of uptake observed in the in vitro specificity test correlated to the different levels of HER3 expression, but no differences were observed in the cellular processing of the different conjugates. While low internalization rate seems to be characteristic for anti-HER3 affibody molecules, the observed rate is considerably lower than for the gallium-68 and indium-111-labeled analogs [26,31,43]. It could be speculated that cobalt-efflux mechanisms are triggered, which in addition to the already slow internalization of (HE)_3_-Z_HER3_, partly affect the level of the internalized fraction [27]. Presence of cobalt ions can be toxic because cobalt competes with biologically essential metal ions, for example iron [44]. Thus, exposure to cobalt could promote the activity of cobalt efflux mechanisms, which seem to be regulated directly by the concentration of cobalt ions [44,45]. Regardless, we also observed continuing increase in the total uptake with time, which might be a product of the strong binding affinity and de novo formation of receptors, which has been reported before [46].

The general biodistribution of [^57^Co]Co-(HE)_3_-Z_HER3_-X was in agreement with the known pattern for affibody molecules and previously reported results for [^57^Co]Co-(HE)_3_-Z_HER3_-NOTA [27]. The affibody conjugates were eliminated quickly via the kidneys and uptake was observed in mErbB3-expressing organs. Even though the biodistribution followed the general known pattern, there were clear differences in tumor targeting and uptake of the affibody–chelator conjugates in normal organs and tissues.

Somewhat surprisingly, the DOTAGA-containing conjugate with the most negative chelator complex (−2) was excreted faster than the other conjugates and had the lowest uptake in mErbB3-positive tissue (except liver) and in the tumor (<1% ID/g). Furthermore, the uptake of this conjugate was blockable in the mErbB3-expressing organs (liver and intestines), but not in tumors. [^57^Co]Co-(HE)_3_-Z_HER3_-DOTAGA was, therefore, considered inferior to the other conjugates. The DOTAGA-conjugated variant also showed unfavorable biodistribution when labeled with gallium-68 [43]. Both of these findings, however, contradict reported results from ^111^In-labeled analogs, where the DOTAGA-conjugated variant had high and stable uptake in tumors and the lowest hepatic uptake providing the best imaging contrast [26].

The distinct biodistribution patterns for the radiometals might be explained by differences in charge and size of the metal–chelator complexes. The ionic radii of cobalt and gallium are similar, and they should thus form comparable geometric complexes. Indium has a larger ionic radius and indium–chelator complexes are, therefore, of different geometry and indium may prefer tetraaza ligands, such as DOTAGA [47,48]. Furthermore, cobalt engages in the radiolabeling in a divalent state, compared to trivalent gallium and indium. As a result, the cobalt–chelator complexes have a more negative charge than their gallium and indium counterparts (e.g., the cobalt–DOTAGA complex has a charge of −2, whereas Ga- and In-DOTAGA complexes have a charge of −1). These differences might explain the different biodistribution patterns of conjugates labeled with different isotopes.

NODAGA- and DOTA-containing conjugates with a single negative charge had similar biodistribution. They had the highest uptake in all organs, except blood. In blood, their activity concentration was significantly lower than for [^57^Co]Co-(HE)_3_-Z_HER3_-NOTA both 3 and 24 h pi. However, the quick tumor washout makes [^57^Co]Co-(HE)_3_-Z_HER3_-NODAGA unfavorable, particularly since the aim was to select a variant for later imaging.

Both [^57^Co]Co-(HE)_3_-Z_HER3_-NOTA and [^57^Co]Co-(HE)_3_-Z_HER3_-DOTA are arguably the most suitable variants at 3 h pi. Regardless, neither variant was able to outperform the gallium-68-labeled affibody molecule [^68^Ga]Ga-(HE)_3_-Z_HER3_-NODAGA at this time point. For the later time point, [^57^Co]Co-(HE)_3_-Z_HER3_-DOTA provided the highest tumor-to-background ratios overall. Remarkably, tumor-to-liver ratio reached 1.6, which, to our knowledge, is the highest ratio published for any single HER3-targeting agent to date. [^57^Co]Co-(HE)_3_-Z_HER3_-NOTA reached a ratio of 1.0 ± 0.2 in this study which is similar to a previously reported value of 1.26 ± 0.05 for the same conjugate [27]. [^89^Zr]Zr-DFO-Z_HER3:8698_ 3 h pi reached a tumor-to-liver ratio of 1.18 ± 0.13. However, some release of the ^89^Zr label was observed in vivo, resulting in a two-fold decrease in tumor uptake after 24 and, therefore, a decrease in tumor-to-liver contrast [22]. Compared with [^111^In]In-Z_HER3_-DOTAGA [26], tumor uptake of [^57^Co]Co-(HE)_3_-Z_HER3_-DOTA was slightly lower (3.4 ± 0.5 vs. 2.4 ± 0.4% ID/g at 24 h), but tumor-to-liver contrast of [^57^Co]Co-(HE)_3_-Z_HER3_-DOTA was higher at both time points, providing another argument for using cobalt-55 as a long-lived radiolabel for PET imaging. Further improvement of imaging contrast might be achieved thorough optimization of the injected protein dose in a phase I/II clinical study similar to studies performed for anti-HER2 affibody molecules [24].

Based on the findings described above, we then investigated the potential benefit of delayed imaging by directly comparing [^57^Co]Co-(HE)_3_-Z_HER3_-DOTA with [^68^Ga]Ga-(HE)_3_-Z_HER3_-NODAGA. [^68^Ga]Ga-(HE)_3_-Z_HER3_-NODAGA was previously determined to be the most favorable variant for imaging of HER3 expression using gallium-68 [31]. [^57^Co]Co-(HE)_3_-Z_HER3_-DOTA (24 h pi) showed a significantly lower concentration of activity in blood and in the liver than gallium-labeled conjugate (3 h pi), while tumor uptake was in the same range. Thus, 24 h pi, this resulted in significantly higher tumor-to-liver and tumor-to-lung ratios for [^57^Co]Co-(HE)_3_-Z_HER3_-DOTA. Particularly in liver, the ratio was 1.6-fold higher than with ^68^Ga label, which is important, since the liver is a common metastatic site in many cancers.

While gallium-68 is produced by widely available ^68^Ga/^68^Ge generators and used in clinics, cobalt-55 is not yet in clinical routine. However, production of cobalt-55 is possible using medical cyclotron [34,35,49] and the longer half-life of cobalt-55 compared to gallium-68 would potentially allow transport to neighboring hospitals without cyclotron access. Therefore, we consider cobalt-55 a promising radioisotope for next-day PET imaging.

In summary, [^57^Co]Co-(HE)_3_-Z_HER3_-DOTA was selected as the best radiocobalt-labeled variant, providing the highest reported tumor-to-liver contrast for HER3-targeting imaging agents thus far. [^57^Co]Co-(HE)_3_-Z_HER3_-DOTA also showed superior tumor-to-liver contrast at a later time point in comparison with the short-lived PET tracer, [^68^Ga]Ga-(HE)_3_-Z_HER3_-NODAGA. We, therefore, conclude that [^57^Co]Co-(HE)_3_-Z_HER3_-DOTA might be a promising alternative for later-time-point PET imaging of HER3 expression.

## 4. Materials and Methods

Cobalt-57 (in form of [^57^Co]CoCl_2,_ (t_1/2_ = 272 d, convenient photons with energies 122 keV (85%), 136 keV (11%) was purchased from JSC-Isotope (JSC-Isotope, Moscow, Russia). Gallium-68 was eluted from a ^68^Ga/^68^Ge generator (Cyclotron Co. Obninsk, Russia) with 0.1 M HCl. Human cancer cell lines BxPC-3 (pancreatic cancer) and DU145 (prostate cancer) were purchased from ATCC (via LGC Promochem, Borås, Sweden) and maintained in RPMI-1640 culture media (Biochrom, Berlin, Germany) supplemented with 10% fetal bovine serum (Merck, Germany), 1% L-Glutamine and 1% Penicillin-Streptomycin (Biochrom, Berlin, Germany). Activity was measured using a 3 inch NaI(Tl) detector (1480 Wizard; Wallac Oy, Turku, Finland).

Affibody molecules (HE)_3_-Z_HER3:08698_-NOTA, (HE)_3_-Z_HER3:08698_-NODAGA, (HE)_3_-Z_HER3:08698_-DOTA, (HE)_3_-Z_HER3:08698_-DOTAGA (further denoted as (HE)_3_-Z_HER3_-X, X = NOTA, NODAGA, DOTA, DOTAGA) were produced in BL21*(DE3) *E. coli* (Escherichia coli) (Thermo Fisher Scientific, Waltham, MA, USA) in an overnight culture at 25 °C and according to methods previously described [31].

Data is presented as the average ± standard deviation if not stated otherwise. Statistical significance (*p* < 0.05) was analyzed with unpaired, two-tailed t-test for the in vitro experiments and in vivo specificity test. One-way ANOVA with post-hoc t-test including correction for multiple comparisons with Bonferroni (GraphPad Prism version 7.03, GraphPad Software, San Diego, CA, USA) was used to test statistical significance between the different (HE)_3_-Z_HER3_-X conjugates in the biodistribution. Two-tailed t-test was used for comparison between [^57^Co]Co-(HE)_3_-Z_HER3_-DOTA and [^68^Ga]Ga-(HE)_3_-Z_HER3_-NODAGA.

### 4.1. Radiolabeling of (HE)_3_-Z_HER3_-X and Stability of Labeled Conjugates

For labeling, 10 µg (HE)_3_-Z_HER3_-X was dissolved in 55 µL sodium acetate (0.2 M, pH 5.5) and incubated with 7.5–14 MBq [^57^Co]CoCl_2_ for 45 min at 60 °C. Instant thin-layered liquid chromatography (ITLC) was used to determine the labeling yields. For analysis, a sample of the radiolabeling mixture was applied to silica gel-impregnated glass micro-fiber chromatography paper (Agilent Technologies, Santa Clara, CA, USA). Samples were eluted with citric acid (0.2 M, pH 2) and scanned with the Cyclone Storage Phosphor System, and OptiQuant image analysis software (PerkinElmer, Waltham, MA, USA) was used to determine the labeling yield.

To test the in vitro stability of [^57^Co]Co-(HE)_3_-Z_HER3_-X, 1 µg of radiolabeled compound was incubated in PBS or 500-fold molar excess of EDTA for 24 h at room temperature. Release of the radiolabel was analyzed with ITLC.

(HE)_3_-Z_HER3_-NODAGA was labeled with gallium-68 according to the method previously described [31]. In brief, 25 µg of (HE)_3_-Z_HER3_-NODAGA was incubated with 300 µL ascorbic acid (1 M, pH 3.6) and 200 µL of gallium-68 eluate (150 MBq) for 15 min at 85 °C and thereafter purified with NAP-5 size-exclusion columns (GE Healthcare, Uppsala, Sweden).

### 4.2. In Vitro Characterization of [^57^Co]Co-(HE)_3_-Z_HER3_-X

HER3-expressing cell lines BxPC-3 (pancreatic cancer) and DU145 (prostate cancer) were used to study the in vitro properties of [^57^Co]Co-(HE)_3_-Z_HER3_-X. Experimental protocols were validated previously for HEr2-binding affibody molecules [50]. Cells were seeded one day before the experiments.

For the in vitro specificity assay, HER3 receptors were pre-saturated by addition of 50 nM unlabeled anti-HER3 affibody. Then, 0.1 nM of [^57^Co]Co-(HE)_3_-Z_HER3_-X was added and the cells were incubated for 1 h at 37 °C. After incubation, the cells were collected, and samples were measured for activity content.

To study the cellular processing of [^57^Co]Co-(HE)_3_-Z_HER3_-X in BxPC-3 and DU145 cell lines, cells were continuously incubated for up to 24 h with 0.1 nM of the labeled constructs at 37 °C. At pre-determined timepoints (1 h, 2 h, 4 h, 8 h, 24 h) the membrane-bound activity was collected after 5 min incubation with 0.2 M glycine buffer with 0.15 M NaCl, 4 M Urea, pH 2, on ice. Thereafter, samples were incubated with 1 M NaOH for 30 min at 37 °C to collect the internalized activity. Sum of the membrane-bound and internalized fraction was considered total cell-associated activity.

Specific binding and cellular processing of [^68^Ga]Ga-(HE)_3_-Z_HER3_-NODAGA has previously been investigated [31].

### 4.3. Measurement of HER3 Receptor Expression in BxPC-3 and DU145 Cells

Quantification of receptor expression was performed similar to [51]. Briefly, cells were incubated with 5 nM [^57^Co]Co-(HE)_3_-Z_HER3_-NOTA for 4 h at 4 °C. To account for unspecific binding, receptors in one cell sample were pre-saturated with 500 nM unlabeled anti-HER3 affibody. After incubation, cells were collected, counted and measured for activity content. Under the assumption of receptor saturation, the number of receptors per cell was calculated based on the amount of cell-bound [^57^Co]Co-(HE)_3_-Z_HER3_-NOTA molecules.

### 4.4. Real Time Measurement of Binding Kinetics

A Ligand Tracer yellow instrument (Ridgeview Instruments AB) was used to measure the binding kinetics (k_a_, k_d_, K_D_) of [^57^Co]Co-(HE)_3_-Z_HER3_-X on BxPC-3 cells in real time [52]. Three million cells were seeded in a designated area of a 10 cm petri dish one day prior to the experiment. For the measurement, the dishes were placed in the inclined rotating holder and [^57^Co]Co-(HE)_3_-Z_HER3_-X was added in several concentrations ranging from 0.2 to 3 nM to measure the association rate. The concentration was increased stepwise when the previous concentration had reached equilibrium. To measure the dissociation rate, the radioactive solution was replaced with cell culture media after the highest concentration reached equilibrium. Data was analyzed with TraceDrawer software (Ridgeview Instruments AB) and association constant (k_a_), dissociation constant (k_d_) and equilibrium dissociation constant (K_D_) were computed using a 1:1 kinetic binding model.

### 4.5. In Vivo Specificity Test and Biodistribution of [^57^Co]Co-(HE)_3_-Z_HER3_-X

Animal experiments were performed in compliance with the national legislation for animal welfare and approved by the Ethics Committee for Animal Research in Uppsala, Sweden (ethical permit number C 5/16 approved 26-02-2016).

Female Balb/c nu/nu mice with BxPC-3 xenografts were intravenously injected with 2 µg (30 kBq) [^57^Co]Co-(HE)_3_-Z_HER3_-X or 2 µg (300 kBq) [^68^Ga]Ga-(HE)_3_-Z_HER3_-NODAGA. Xenograft model was chosen based on the HER3 expression and injected protein dose based on our previous results [16]. Animals were euthanized 3 and 24 h pi by injection of Ketalar–Rompun solution (10 mg/mL Ketalar and 1 mg/mL Rompun; 20 μL solution/gram of body weight). Samples of blood, salivary glands, lung, liver, stomach, small intestine, tumor, spleen, kidney, muscle and bone were collected, weighed and measured for activity content. To test in vivo specificity of [^57^Co]Co-(HE)_3_-Z_HER3_-X the injected protein dose was increased to 70 µg using unlabeled Z_HER3_. Animals were sacrificed 3 h pi, and samples were collected and analyzed according to the protocol described above.

At the time of the experiments, the average mouse weight was 18 ± 1 g and average tumor weight was 0.09 ± 0.08 g.

### 4.6. Imaging of HER3-Expressing Bxpc-3 Xenografted Mice

Balb/c nu/nu mice with BxPC-3 xenograft were injected with 2 µg (1–1.9 MBq) [^57^Co]Co-(HE)_3_-Z_HER3_-X and whole-body SPECT/CT scans were acquired 3 and 24 h pi using nanoScan SPECT/CT (Mediso Medical Imaging Systems Ltd., Budapest, Hungary). Mice were euthanized before imaging. CT was acquired at the following parameters: 50 keV energy peak, 670 μA, 480 projections, 5.26 min acquisition time; SPECT was carried out using a ^57^Co energy peak of 122.1 keV, window width of 20%, and a matrix of 256 × 256, and was acquired for 1 h. Nucline 2.03 software was used to reconstruct CT data and Tera-Tomo^TM^ 3D SPECT reconstruction technology was used for SPECT data (Mediso Medical Imaging Systems Ltd., Budapest, Hungary).

Whole-body gallium-68 PET/CT imaging was performed 3 h pi according to previously published methods [31]. A BxPC-3 xenografted mouse was injected with 2 µg (7.8 MBq) [^68^Ga]Ga-(HE)_3_-Z_HER3_-NODAGA and imaged 3 h pi after euthanasia.

## Figures and Tables

**Table 1:** Labeling and stability of [^57^Co]Co-(HE)_3_-Z_HER3_-X.

	NOTA *	NODAGA	DOTA	DOTAGA
Radiochemical yield (%)	81 ± 11% (*n* = 6)	99.7 ± 0.2 (*n* = 2)	99.7 ± 0.4 (*n* = 2)	99.3 ± 0.7 (*n* = 2)
% Release in PBS, 24 h, RT	stable	0 ± 0	0 ± 0	0.2 ± 0.3
% Release in human serum, 24 h, 37 °C		0 ± 0	0.4 ± 0.8	0.03 ± 0.05

Radiochemical yield and stability were determined with instant thin-layered liquid chromatography (ITLC). Stability data is expressed as % release. * Labeling and stability test of [^57^Co]Co-(HE)_3_-Z_HER3_-NOTA were previously published by [27]. Purity of [^57^Co]Co-(HE)_3_-Z_HER3_-NOTA was >99% after purification with NAP5 size-exclusion chromatography [27].

**Table 2:** Affinity measurements. Association rate (k_a_), dissociation constant (k_d_) and equilibrium dissociation constant (K_D_) measured on living BxPC-3 cells in real time using Ligand Tracer.

	NOTA (*n* = 3)	NODAGA (*n* = 3)	DOTA (*n* = 4)	DOTAGA (*n* = 3)
k_a_ (1/Ms)	2 × 10^5^ ± 2 × 10^5^	1.19 × 10^5^ ± 0.09 × 10^5^	1.0 × 10^5^ ± 0.3 × 10^5^	1.2 × 10^5^ ± 0.8 × 10^5^
k_d_ (1/s)	1.28 × 10^−5^ ± 0.10 × 10^−5^	1.0 × 10^−5^ ± 0.7 × 10^−5^	2 × 10^−5^ ± 1 × 10^−5^	1.4 × 10^−5^ ± 0.4 × 10^−5^
K_D_ (nM)	0.1 ± 0.1	0.09 ± 0.07	0.2 ± 0.1	0.2 ± 0.1

**Table 3:** Ex vivo biodistribution. Female Balb/c nu/nu mice with HER3-expressing BxPC-3 xenografts were injected with 2 µg [^57^Co]Co-(HE)_3_-Z_HER3_-X (X = NOTA, NODAGA, DOTA, DOTAGA).

Organ	NOTA		NODAGA		DOTA		DOTAGA	
	3 h	24 h	3 h	24 h	3 h	24 h	3 h	24 h
Blood	1.0 ± 0.1 ^a,b,c,^*	0.31 ± 0.04 ^a,b,c,^*	0.61 ± 0.09 ^a,d,^*	0.18 ± 0.02 ^a,^*	0.33 ± 0.04 ^b,d,f,^*	0.14 ±0.02 ^b,f,^*	0.52 ±0.04 ^c,f,^*	0.22 ±0.02 ^c,^*
Salivary Gland	0.9 ± 0.2 ^a,b^	0.6 ± 0.2	1.6 ± 0.4 ^a,e^	1 ± 1	1.5 ± 0.1 ^b,f,^*	0.9 ± 0.1 *	0.64 ±0.09 ^e,f^	0.4 ±0.1
Lung	1.0 ± 0.1 ^a,b,^*	0.41 ± 0.07 *	1.7 ± 0.3 ^a,e,^*	0.47 ± 0.04 *	1.7 ± 0.2 ^b,f,^*	0.6 ± 0.2 ^e,^*	0.8 ± 0.1 ^e,f,^*	0.33 ± 0.04 ^e,^*
Liver	1.56 ± 0.52 ^a,b^	1.1 ± 0.2	3.3 ± 0.8 ^a,^*	1.4 ± 0.2 *	3.7 ± 0.5 ^b,f,^*	1.6 ± 0.2 *	2.4 ± 0.2 ^e,f,^*	1.5 ± 0.2 *
Spleen	0.57 ± 0.04 ^c^	0.6 ± 0.2	0.47 ±0.09 ^e^	0.35 ±0.05 ^e^	0.43 ± 0.05 ^f^	0.42 ± 0.08	0.8 ± 0.1 ^c,e,f^	0.7 ± 0.1 ^e^
Stomach	0.78 ± 0.06 ^a,b,^*	0.35 ± 0.04 ^a,b,^*	1.5 ±0.5 ^a,e,^*	0.65 ± 0.06 ^a,e,^*	1.5 ± 0.3 ^b,f,^*	0.65 ± 0.05 ^b,f,^*	0.6 ± 0.1 ^e,f,^*	0.31 ± 0.05 ^b,f,^*
Small intestine	1.6 ±0.3 ^a,b,^*	0.7 ± 0.1 ^a,b,^*	4 ± 1 ^a,e,^*	1.6 ±0.3 ^a,e,^*	4.6 ± 0.8 ^b,f,^*	2.1 ± 0.04 ^b,f,^*	0.9 ± 0.2 ^e,f^	0.6 ± 0.10 ^e,f^
Kidneys	194 ± 17 *	106 ± 67 ^a,b,^*	279 ± 84	213 ± 7 ^a^	253 ± 54	223 ± 23 ^b,f^	156 ± 27 ^c^	131 ±13 ^f^
Tumor	1.55 ± 0.26 ^a,b^	1.1 ± 0.3 ^b^	2.6 ± 0.6 ^a,e,^*	1.4 ± 0.4 ^d,^*	2.8 ± 0.4 ^b,f^	2.4 ± 0.4 ^b,d,f^	0.9 ± 0.2 ^e,f^	0.8 ± 0.1 ^f^
Muscle	0.19 ± 0.04	0.13 ± 0.04	0.20 ± 0.06 *	0.09 ± 0.02 *	0.19 ± 0.01 *	0.09 ± 0.02 *	0.16 ± 0.02	0.11 ± 0.03
Bone	0.3 ± 0.1	0.25 ± 0.05	0.3 ± 0.1	0.15 ±0.05	0.25 ± 0.06	0.20 ± 0.03	0.28 ± 0.09	0.19 ± 0.07
GI (%ID)	2.4 ± 0.5 ^a,b,^*	1.3 ± 0.2 *	5.0 ± 0.4 ^a,^*	2.8 ± 0.7 *	5.7 ± 0.9 ^b,f,^*	2.7 ± 0.7 *	4.78 ± 0.9^f^	1.13 ±0.2
Body (%ID)	5.9 ± 0.4 ^a,b^	3 ± 2 ^a,b^	10 ± 2 ^a,e,^*	4.5 ± 0.7 ^a,e,^*	9.0 ± 1.0 ^b,f,^*	4.9 ± 0.56 ^b,f,^*	2 ± 0.6 ^e,f,^*	3.1 ±0.3 ^e,f,^*

Data presented as % ID/g and average ± SD of *n* = 4 animals per group. Significant difference (*p* < 0.05) between ^a^: NOTA vs. NODAGA, ^b^: NOTA vs. DOTA, ^c^: NOTA vs. DOTAGA, ^d^: NODAGA vs. DOTA, ^e^: NODAGA vs. DOTAGA, ^f^: DOTA vs. DOTAGA. * Significant difference between 3 and 24 h.

**Table 4:** Tumor-to-organ ratios. Female Balb/c nu/nu mice with HER3-expressing BxPC-3 xenografts were injected with 2 µg [^57^Co]Co-(HE)_3_-Z_HER3_-X (X = NOTA, NODAGA, DOTA, DOTAGA).

Tumor/Organ Ratios	NOTA		NODAGA		DOTA		DOTAGA	
	3 h	24 h	3 h	24 h	3 h	24 h	3 h	24 h
T/Blood	1.5 ± 0.2 ^a,b,^*	3.4 ± 0.6 ^b^	4 ± 1^a,d,e^	8 ± 3^d^	8 ± 1 ^b,d,f,^*	18 ± 5 ^b,d,f^	1.8 ±0.3 ^e,f,^*	3.4 ± 0.5 ^f^
T/Salivary gland	1.8 ± 0.3	1.7 ± 0.3	1.7 ± 0.3	1.3 ± 0.7 ^d^	1.9 ± 0.2*	2.9 ± 0.6 ^d^	1.5 ± 0.3	2.1 ± 0.9
T/Lung	1.6 ±0.3 *	2.6 ± 0.4	1.6 ± 0.6	3 ± 1	1.6 ± 0.4*	4.1 ± 0.9 ^e^	1.1 ±0.1*	2.3 ± 0.4 ^e^
T/Liver	1.06 ± 0.31 ^c^	1.0 ± 0.2 ^b^	0.78 ± 0.09 ^e^	1.0 ± 0.3	0.74 ± 0.08 ^f,^*	1.6 ± 0.3 ^b,f^	0.44 ± 0.09 ^c,e,f^	0.50 ± 0.07 ^f^
T/Spleen	2.7 ± 0.3 ^a,b,^*	1.89 ±0.08 ^b^	6 ± 2 ^a,e^	4 ± 2 ^e^	6 ± 1 ^b,f^	6 ± 1 ^f^	1.2 ± 0.2 ^e,f^	1.2 ± 0.3 ^e,f^
T/Stomach	2.0 ± 0.2 *	3.1 ± 0.8	1.7 ±0.4	2.1 ± 0.7	2 ± 0.2 *	3.8 ± 0.9	1.6 ± 0.5 *	2.5 ± 0.4
T/Small intestine	1.0 ± 0.2 ^b,^*	1.6 ± 0.2	0.6 ± 0.2	0.9 ± 0.4	0.60 ± 0.05 ^b,f^	1.3 ± 0.6	1.0 ± 0.1 ^e,f^	1.3 ± 0.2
T/Muscle	8 ± 2 ^a,b^	8 ± 1 ^b^	14 ± 4 ^a,e^	17 ± 7 ^d,e^	15 ± 1 ^b,f,^*	28 ± 4 ^b,d,f^	6 ± 1 ^e,f^	7 ± 2 ^e,f^
T/Bone	6 ± 3	4.3 ± 0.4 ^b^	10 ± 3	10 ±7	11 ± 2 ^f^	12 ± 3 ^e^	5 ± 3 ^f^	4 ± 1 ^e^

Data presented as the average ± SD of *n* = 4 animals per group. Significant difference (*p* < 0.05) between ^a^: NOTA vs. NODAGA, ^b^: NOTA vs. DOTA, ^c^: NOTA vs. DOTAGA, ^d^: NODAGA vs. DOTA, ^e^: NODAGA vs. DOTAGA, ^f^: DOTA vs. DOTAGA; * significant difference to 24 h.

**Table 5:** Positron-emitting nuclides with potential for imaging at 24 h after injection (https://www.nndc.bnl.gov/nudat2/indx_dec.jsp).

Nuclide	Half-Life (Hour)	Mode of Decay	Mean Positron Energy (keV)	Principal Photon Emissions
^55^Co	17.5	β^+^ 76% EC 24%	570	511 (152%), 477 (20.2%), 931 (75%), 1317 (7.1%), 1408 (16.9%)
^64^Cu	12.7	β^+^ 17.6% β^−^ 37% EC 24%	278	511 (35.2%), 1346 (0.5%)
^66^Ga	9.49	β^+^ 57% EC 43%	1750	511 (114%), 834 (5.9%), 1039 (37.0%), 2752 (22.7%)
^86^Y	14.7	β^+^ 31.9 %EC 67%	660	511 (66%), 443 (16.9%), 628 (32.6%), 646 (9.2%), 703 (15.4%), 777 (22.4%), 1077 (82.5%), 1153 (30.5%), 1854 (17.2%), 1920 (20.8%)
^90^Nb	14.6	β^+^ 51.2% EC 49.8%	660	511 (102%), 141 (66.8%), 1129 (92.7%), 2186 (18%), 2318 (82%)
^152^Tb	17.5	β^+^ 20.3% EC 79.7%	1140	511 (40.6%), 271 (9.5%), 344 (63.5%), 586 (9.2%), 779 (5.5%)
^76^Br	16.2	β^+^ 55% EC 45%	1188	511 (110%), 657 (16%), 1853 (14.7%), 2792 (5.6%), 2950 (7.4%)

## Supplementary Materials

Supplementary materials can be found at https://www.mdpi.com/1422-0067/21/6/1972/s1.

## Abbreviations

CT	Computed tomography
DOTA	1,4,7,10-tetraazacyclododecane-1,4,7,10-tetraacetic acid
DOTAGA	1,4,7,10-tetraazacyclododecane,1-(glutaric acid)-4,7,10-triacetic acid
EDTA	Ethylenediaminetetraacetic acid
EPR effect	Enhanced permeability and retention effect
ESP	Engineered scaffold protein
HER	Human epidermal growth factor receptor
ITLC	Instant thin-layered liquid chromatography
MIP	Maximum intensity projection
MRI	Magnetic resonance imaging
NOTA	1-(1,3-carboxypropyl)-4,7-carboxymethyl-1,4,7-triazacyclononane
NODAGA	1,4,7-triazacyclononane-N,N′,N″-triacetic acid
PBS	Phosphate buffer saline
PET	Positron emission tomography
RP-HPLC	Reverse-phase high-performance liquid chromatography
SPECT	Single-photon emission tomography
