# Supplementary material for: Benefit of Later-Time-Point PET Imaging of HER3 Expression Using Optimized Radiocobalt-Labeled Affibody Molecules
Source: Int J Mol Sci. 2020 Mar 13;21(6):1972. doi: 10.3390/ijms21061972 (PMC7139902; doi:10.3390/ijms21061972)
Supplement: Supplementary file 1 [file ijms-21-01972-s001.pdf]

Benefit of later time point PET imaging of HER3 expression using optimized radiocobalt-labeled affibody molecule

Sara S. Rinne, Charles Dahlsson Leitao, Zahra Saleh-nihad, Bogdan Mitran, Vladimir Tolmachev, Stefan Ståhl, John Löfblom and Anna Orlova

Supplementary Data

Table 1. Biodistribution data for [^{68}Ga]Ga-(HE)$_3$-Z$_{HER3}$-NODAGA (3 h pi) in BxPC-3 xenografted Balb/c nu/nu mice as %ID and tumor-to-organ ratios.

Organ	% ID/g			Tumor to Organ Ratio		
	**^{68}Ga-(HE)$_3$-Z$_{HER3}$-NODAGA (3 h pi)**	**[^{57}Co]Co-(HE)$_3$-Z$_{HER3}$-DOTA (3 h pi)**	**[^{57}Co]Co-(HE)$_3$-Z$_{HER3}$-DOTA (24 h pi)**	**^{68}Ga-(HE)$_3$-Z$_{HER3}$-NODAGA (3 h pi)**	**[^{57}Co]Co-(HE)$_3$-Z$_{HER3}$-DOTA (3 h pi)**	**[^{57}Co]Co-(HE)$_3$-Z$_{HER3}$-DOTA (24 h pi)**
Blood	0.18 ± 0.01 [a,b]	0.33 ± 0.04	0.14 ±0.02	12 ± 2 [a]	8 ± 1	18 ± 5
Salivary gland	0.7 ± 0.1 [a]	1.5 ± 0.1	0.9 ± 0.1	3.1 ± 0.6 [a]	1.9 ± 0.2	2.9 ± 0.6
Lung	0.81 ± 0.08 [a]	1.7 ± 0.2	0.6 ± 0.2	2.8 ± 0.3 [a,b]	1.6 ± 0.4	4. 1 ± 0.9
Liver	2.2 ± 0.1 [a,b]	3.7 ± 0.5	1.6 ± 0.2	1.0 ± 0.1 [a,b]	0.74 ± 0.08	1.6 ± 0.3
Spleen	0.29 ± 0.04 [a,b]	0.43 ± 0.05	0.42 ± 0.08	8 ± 2	6 ± 1	6 ± 1
Stomach	0.8 ± 0.02 [a,b]	1.5 ± 0.3	0.65 ± 0.05	2.8 ± 0.6	2 ± 0.2 *	3.8 ± 0.9
Small Intestine	1.7 0.04 [a]	4.6 ± 0.8	2.1 ± 0.04	1.3 ± 0.2 [a]	0.60 ± 0.05	1.3 ± 0.6
Kidneys	271 ± 36	253 ± 54	223 ± 23	0.008 ± 0.001	0.011 ± 0.002	0.011 ± 0.003
Tumor	2.3 ± 0.2	2.8 ± 0.4	2.4 ± 0.4	-	-	-
Muscle	0.10 ± 0.04 [a]	0.19 ± 0.01	0.09 ± 0.02	26 ± 12	15 ± 1	28 ± 4
Bone	0.3 ± 0.2	0.25 ± 0.06	0.20 ± 0.03	8 ± 5	11 ± 2	12 ± 3 [e]

Statistical significant differences ($p < 0.05$) in uptake and tumor-to-organ ratios of [^{68}Ga]Ga-(HE)$_3$-Z$_{HER3}$-NODAGA (3 h pi) and [^{57}Co]Co-(HE)$_3$-Z$_{HER3}$-DOTA at [a] 3 h pi and [b] 24 h pi was determined with unpaired, two-tailed t-test.

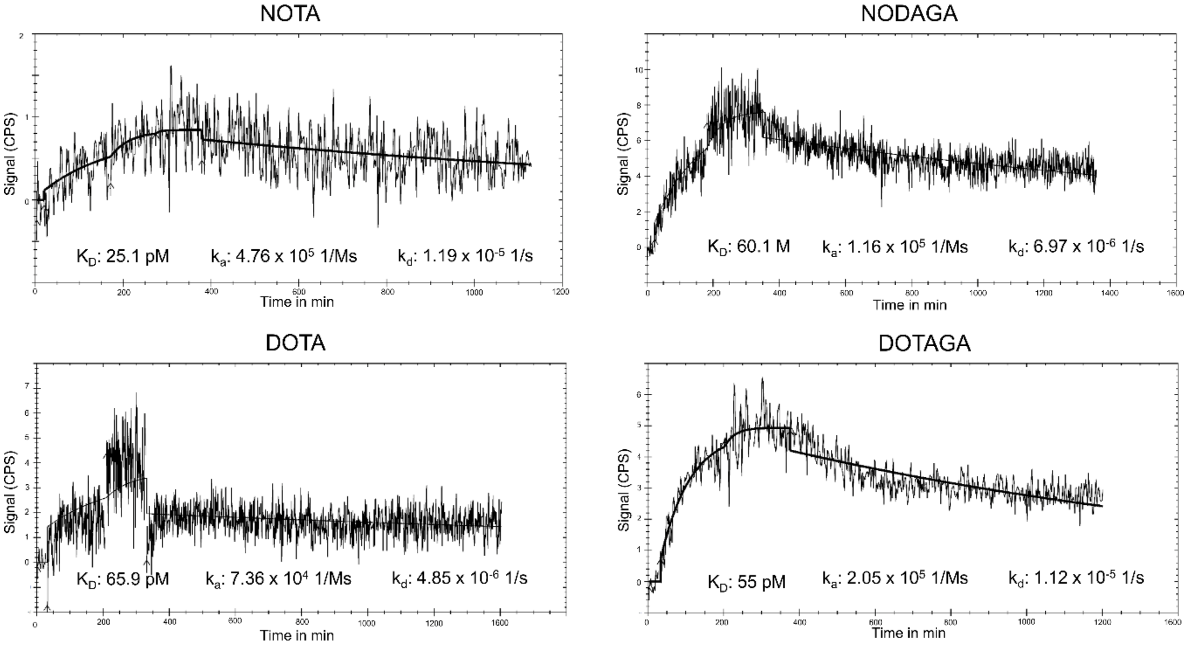

Figure 1. Representative Ligand Tracer Curves. Binding Kinetics of ^{57}Co-(HE)$_3$-Z$_{HER3}$-X were measured in real time on HER3 expressing BxPC-3 cells using Ligand Tracer yellow instruments (Ridgeview Instruments AB).

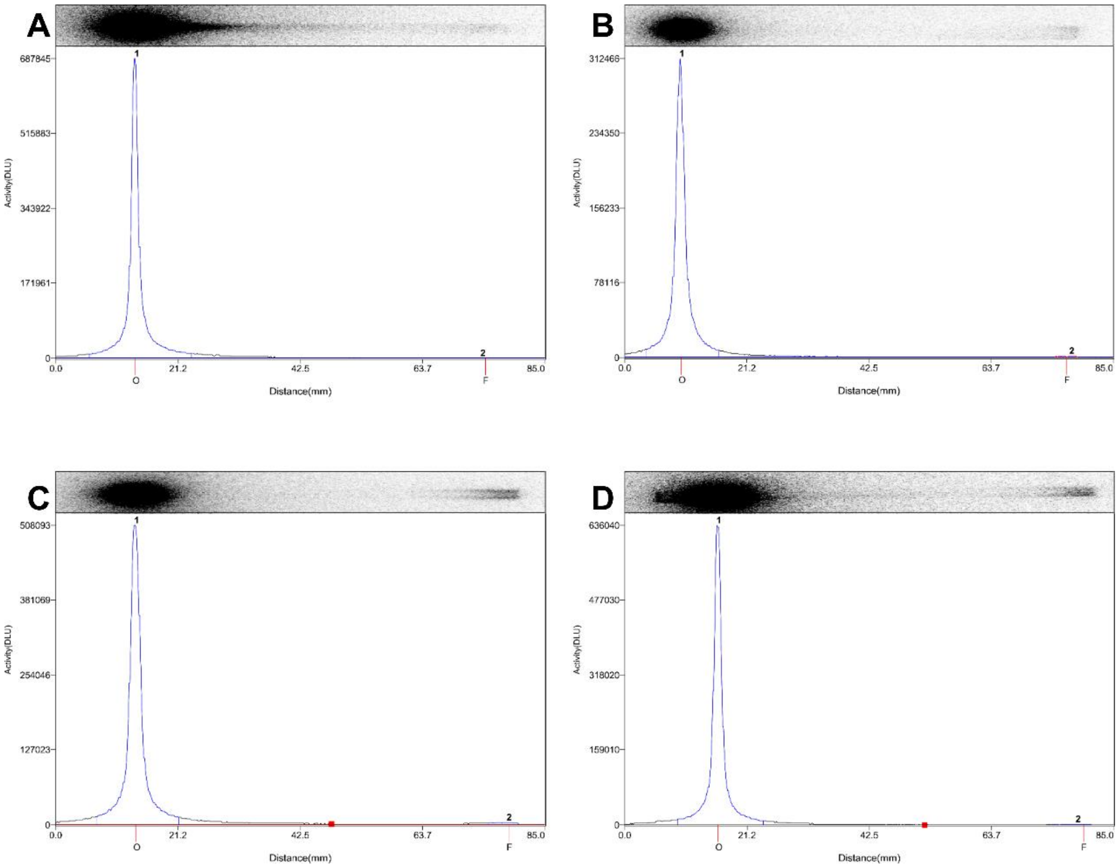

Figure 2. Representative ITLC traces of the radiolabeled products. (**A**) ^{57}Co-(HE)$_3$-Z$_{HER3}$-NOTA (**B**) ^{57}Co-(HE)$_3$-Z$_{HER3}$-NODAGA (**C**) ^{57}Co-(HE)$_3$-Z$_{HER3}$-DOTA (**D**) ^{57}Co-(HE)$_3$-Z$_{HER3}$-DOTAGA. Labeling was performed in ammonium acetate buffer (0.2 M, pH 5.5) for 45 minutes at 60 °C. Samples were taken from the labeling mixture and analyzed by ITLC using citric acid as the mobile phase. Radiolabeled conjugates remain at the application point (**O**) and free cobalt migrates to the front (**F**).
